# Unlocking HIV-1 Env: implications for antibody attack

**DOI:** 10.1186/s12981-017-0168-5

**Published:** 2017-09-12

**Authors:** Jonathan Richard, Shilei Ding, Andrés Finzi

**Affiliations:** 1 Centre de Recherche du CHUM (CRCHUM), 900 St-Denis Street, Tour Viger, Montréal, QC H2X 0A9 Canada; 2 Department of Microbiology, Infectiology and Immunology, Université de Montréal, Montreal, QC Canada; 3 Department of Microbiology and Immunology, McGill University, Montreal, QC Canada

**Keywords:** HIV-1, Env, gp120, ADCC, Nef, Vpu, BST-2, CD4, CD4-mimetics, RV144

## Abstract

Collective evidence supporting a role of Antibody-Dependent Cell-Mediated Cytotoxicity (ADCC) in controlling HIV-1 transmission and disease progression emerged in the last few years. Non-neutralizing antibodies (nnAbs) recognizing conserved CD4-induced epitopes on Env and able to mediate potent ADCC against HIV-1-infected cells exposing Env in its CD4-bound conformation have been shown to be present in some RV144 vaccinees and most HIV-1-infected individuals. HIV-1 evolved sophisticated strategies to decrease exposure of this Env conformation by downregulating CD4 and by limiting the overall amount of cell-surface Env. In this review, we will summarize our contribution to this rapidly evolving field, discuss how structural properties of HIV-1 Env might have contributed to the modest efficacy of the RV144 trial and how we recently used this knowledge to develop new strategies aimed at sensitizing HIV-1-infected cells to ADCC mediated by easy to elicit nnAbs.

## Background

Neutralizing antibodies (NAbs) are generally central components of a protective vaccine-induced immune response. While design of immunogens able to elicit broadly reactive Nabs (bNAbs) remains a major goal of HIV-1 vaccine development, no HIV-1 vaccine candidate has fulfilled this goal [1]. To date, only one anti-HIV-1 vaccine trial, the RV144 trial conducted in Thailand, presented a modest (31.2%) efficacy in preventing HIV-1 infection [2]. Rather than bNAbs or CD8+ T cell response, protection was associated with the presence of anti-Env ADCC-mediating Abs in a subset of individuals with low plasma anti-Env IgA titer [3]. Accordingly, non-neutralizing antibodies (nnAbs) with potent ADCC activity were isolated from some RV144 vaccinees [4]. These findings suggested that ADCC-mediating Abs may have contributed to the partial protection observed in the RV144 trial and renewed interest in the mechanisms of recognition of these antibodies.

## HIV-1 reduces Env-CD4 interaction to prevent ADCC

Besides being exposed at the surface of viral particles, the Env trimer represents the only virus-specific target at the surface of infected cells. We showed that interaction of Env with the viral receptor CD4 at the surface of the same HIV-1-infected cell is critical for the exposure of Env epitopes targeted by ADCC-mediating Abs [5]. Strikingly, we demonstrated that multiple Abs with potent ADCC activity, including those isolated from RV144 vaccinees, preferentially target infected cells exposing Env in its CD4-bound conformation. Importantly, we observed that these antibodies are not uncommon since we observed that sera from a large number of HIV-1-infected individuals, at different stages of disease progression, contain a high prevalence of Abs that recognizes CD4-induced (CD4i) Env epitopes able to mediate ADCC responses [6]. We further characterized the specificity of these Abs for their ability to mediate ADCC and found that anti-cluster A Abs, which recognize layer 1 and 2 of the gp120 inner domain, have a unique ability to eliminate infected cells exposing Env in its CD4-bound conformation [7, 8]. These nnAbs recognize transitional epitopes located in the inner domain of the gp120 subunit that are normally buried in the unbound Env trimer. These epitopes are commonly detected by Abs present in sera from HIV-1-infected individuals [6, 7, 9]. Accordingly, we demonstrated that a highly conserved tryptophan at position 69 of the gp120 inner domain, which plays a crucial role for Env trimer stability and its ability to transition to the CD4-bound conformation, is also critical for ADCC responses mediated by anti-cluster A Abs and HIV+ sera [7].

**Fig. 1:**
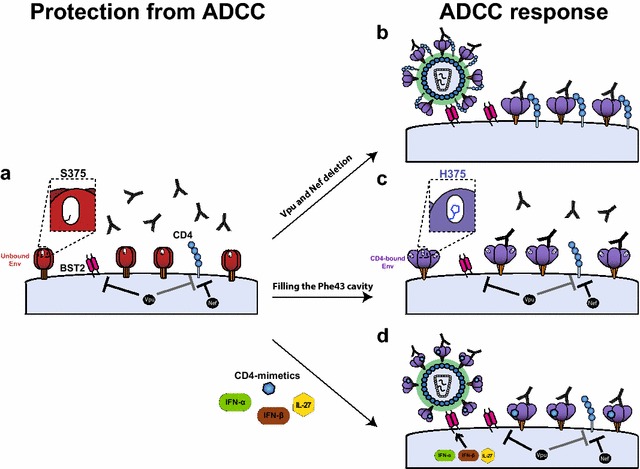
Unlocking HIV-1 Env for antibody attack. ADCC-mediating Abs present in some RV144 vaccinees and sera from HIV-1-infected individuals preferentially recognize Env in its CD4-bound conformation (Env in the unbound conformation is shown in *red* and in the CD4-bound conformation is shown in *purple*). To limit the exposure of Env CD4i epitopes, HIV-1 has evolved protective mechanisms to counteract the host restriction factor BST-2 through Vpu-mediated downregulation. It also downregulates CD4 via Nef and Vpu activities (**a** Nef and Vpu accessory proteins are shown in *black*). Deletion of Vpu and Nef results in accumulation of Env-CD4 complexes at the cell-surface, enhancing the susceptibility of HIV-1-infected cells to ADCC (**b**). Flanking the Phe43 cavity, the identity of residue 375 modulates the transition of Env to the CD4-bound conformation. The presence of a small amino acid such as serine (S375) at this position keeps Env in its unbound “closed” conformation (**a**), thus preventing the exposure of CD4i ADCC-mediating epitopes. The presence of a larger residue within the Phe43 cavity, such as the naturally-occurring histidine at position 375 (H375) in CRF01_AE strains, shifts Env conformation to a state closer to the CD4-bound state, enhancing susceptibility of infected cells to ADCC mediated by HIV+ sera and easy-to-elicit antibodies (**c**). Small CD4-mimetics (CD4mc) sensitize HIV-1-infected cells to ADCC mediated by HIV+ sera by forcing Env to sample its CD4-bound conformation. Type I IFNs (IFNα is shown in *green* and IFNβ in *brown*) or IL-27 (shown in *yellow*) treatment, through upregulation of BST-2, boosts the ability of CD4mc to sensitize HIV-1-infected cells to ADCC by increasing the amounts of Env able to interact with CD4mc at the cell surface (**d**)

Our finding suggest that antibodies elicited in the majority of HIV-1-infected individuals do have the potential to eliminate infected cells by ADCC, but preferentially target Env in its CD4-bound conformation. However, the virus limits the exposure of this Env conformation and therefore protects infected cells from ADCC. We and others found that HIV-1 efficiently limits Env-CD4 interaction and the exposure of CD4i Env epitope by downregulating CD4 and the restriction factor BST-2 (also known as Tetherin/CD317/HM1.24) from the surface of infected cells. First, Vpu-mediated BST-2 downregulation prevents accumulation of nascent virions on the surface of infected cells [5, 10, 11]. Second, Nef and Vpu-mediated CD4 downregulation effectively prevents cell-surface Env-CD4 interaction [5]. These findings suggest that these accessory proteins play a major role in reducing the susceptibility of HIV-1-infected cells to ADCC (Fig. 1).

## Influence of the Phe43 cavity on Env conformation and ADCC

In addition to Vpu and Nef action, structural features of HIV-1 Env also influence the sensitivity of HIV-1 to ADCC. The Phe43 cavity, located at the interface of the inner and outer domains of gp120, allows the engagement with CD4 via its Phe43 residue [12] and modulates the propensity of Env that sample the CD4-bound conformation [13]. Substitution of the well-conserved group M serine at position 375 by a larger residue such as tryptophan or histidine was found to fill the Phe43 cavity and result in the spontaneous sampling of a conformation closer to the CD4-bound state [13]. Accordingly, we showed that filling this cavity with a histidine or tryptophan residue increased the susceptibility of HIV-1-infected cells to ADCC [14] (Fig. 1c). Surprisingly, while residue S375 is well-conserved among group M HIV-1 isolates, the predominant CRF01_AE strain in Thailand where the RV144 trial took place has a naturally-occurring histidine at this position (H375). Interestingly, H375 was recently shown to be important for CD4 binding in this strain [15] and its substitution by a serine (H375S) substantially reduces ADCC against CRF01_AE-infected cells mediated by Abs isolated from RV144 vaccinees [14]. While a functional coevolution between the Phe43 cavity and the gp120 inner domain layers appears to compensate for the presence of H375 in CRF01_AE Envs [15], our results raise the intriguing possibility that this unique H375 polymorphism present in the circulating strains in Thailand might have contributed to the efficacy of the RV144 trial by naturally exposing CD4i ADCC-mediating epitopes. Although our results suggest that H375 might represent a point of vulnerability for the virus in vaccine settings, they do not necessarily imply that viral strains carrying the H375 polymorphism would be better controlled once the infection has been established. Supporting this, a recent study showed that filing the Phe43 cavity by residue 375 substitutions in simian-human immunodeficiency viruses (SHIV) enhanced viral replication in rhesus macaques [16].

## Mimicking Env-CD4 interaction to sensitize HIV-1-infected cells to ADCC

The majority of circulating HIV-1 strains worldwide encodes functional Vpu and Nef proteins and express Env with “empty” Phe43 cavities (S375), which limit exposure of epitopes recognized by CD4i ADCC-mediating antibodies. Interestingly, we recently described a new strategy to overcome these protective mechanisms and sensitize HIV-1-infected cells to ADCC by modulating Env conformation using small CD4-mimetic compounds (CD4mc) [17]. These CD4mc engage gp120 within the Phe43 cavity and can induce thermodynamic changes in the Env trimer similar to those observed upon binding by soluble CD4 [18]. Using these CD4mc, we were able to force Env present at the surface of infected cells to sample the CD4-bound conformation and enhance recognition of infected cells by sera, breast milk and cervicovaginal fluids from HIV-1-infected subjects [17]. This approach not only efficiently sensitized cells infected with full-length HIV-1 primary isolates to ADCC mediated by these biological fluids but it also sensitized endogenously infected ex vivo-amplified primary CD4+ T cells to ADCC mediated by autologous sera and autologous effector cells [17]. We rationalized that the effect of CD4mc on ADCC could be influenced by the amount of Env present at the surface of infected cells where only limited amounts of Env are exposed due to efficient Vpu-mediated BST-2 downregulation [5, 10, 11]. Accordingly, we recently found that BST-2 expression, and its sensitivity to Vpu down modulation, dictate the ability of CD4mc to sensitize HIV-1-infected cells to ADCC by modulating the amount of Env able to engage CD4mc [19]. Interestingly, we found that BST-2 upregulation by IFN-α, IFN-β or IL-27 induces Env accumulation at the cell surface and boosts the ability of CD4mc to sensitize HIV-1-infected cells to ADCC mediated by sera from HIV-1-infected individuals. These results suggest that combination of type 1 IFNs or IL-27 with CD4mc, might represent an attractive approach to target and eliminate HIV-1-infected cells through ADCC (Fig. 1d).

## Molecular understanding of the exposure of vulnerable Env epitopes

We recently characterized the different steps involved in the exposure of ADCC-mediating anti-cluster A epitopes and showed that it requires a sequential opening of the Env trimer [20]. We found that CD4mc synergize with co-receptor binding site (CoRBS) Abs present in HIV+ sera to efficiently expose anti-cluster A epitopes and sensitize HIV-1-infected cells to ADCC. These findings helped to better define the specific Abs that could contribute to eliminate HIV-1-infected cells by ADCC and also provided crucial information for the design of immunogens aimed at generating an efficient ADCC response. Interestingly, both CoRBS and anti-cluster A Abs recognize highly-conserved Env epitopes and therefore such combination of nnAbs might represent a broad and potent approach to unlock HIV-1 Env and sensitize HIV-1-infected cells to ADCC.

## Enhancing neutralization and ADCC activity of vaccine-elicited nnAbs using CD4mc

Recent studies identified different bNAbs that can also mediate ADCC responses [11, 21, 22]. However, elicitation of potent bNAb is relatively rare and occurs after several years of infection [23]. Moreover, elicitation of bNAbs by immunization remains an important challenge, since to date, no immunogen has induced bNAb responses that match those elicited during natural infection. In contrast, CD4i nnAbs are easy to elicit, commonly detected in HIV-1-infected individuals and mediate broad and potent ADCC responses when Env is exposed in its CD4-bound conformation. While vaccine trials in non-human primate (NHP) using various Env immunogens were unable to elicit bNAbs so far, sera from vaccinated NHP using different Env-based immunogens could easily neutralize HIV-1 in the presence of CD4mc. Thus, proving that CD4mc can sensitize HIV-1 Env to neutralization by easy-to-elicit CD4i Abs [24]. Moreover, the same strategy resulted in efficient ADCC elimination of HIV-1-infected cells through ADCC responses [25]. Altogether, these data suggest that combining Env-based immunogens with a small-molecule CD4mc, administered orally or in a microbicide formulation, might be useful as a prophylactic strategy against HIV-1 transmission.

## Conclusions

All the elements required to eliminate HIV-1-infected cells by ADCC are already present in HIV-1-infected individuals. However, HIV-1 evolved multiple mechanisms to protect virus-producing cells from ADCC by reducing Env-CD4 complexes. Importantly, by enhancing both neutralization and ADCC activity of nnAbs, naturally present during HIV-1 infection or elicited upon immunization, CD4mc hold the promise of therapeutic utility in preventing and controlling HIV-1-infection.
